# Current Mechanobiological Pathways and Therapies Driving Spinal Health

**DOI:** 10.3390/bioengineering12080886

**Published:** 2025-08-20

**Authors:** Rahul Kumar, Kyle Sporn, Harlene Kaur, Akshay Khanna, Phani Paladugu, Nasif Zaman, Alireza Tavakkoli

**Affiliations:** 1 Department of Biochemistry and Molecular Biology, University of Miami Miller School of Medicine, Miami, FL 33136, USA; rxk641@miami.edu; 2 Department of Medicine, Norton College of Medicine, 785 E Adams St., Syracuse, NY 13202, USA; spornk@upstate.edu; 3 School of Medicine, University of Massachusetts T.H. Chan School of Medicine, 55 N Lake Avenue, Worcester, MA 01655, USA; harlene.kaur3@umassmed.edu; 4 Sidney Kimmel Medical College, Thomas Jefferson University, 1025 Walnut Street #100, Philadelphia, PA 19107, USA; aya156@students.jefferson.edu (A.K.); phani.paladugu@students.jefferson.edu (P.P.); 5 Brigham and Women’s Hospital, Harvard Medical School, 75 Francis St., Boston, MA 02115, USA; 6 Human-Machine Perception Laboratory, Department of Computer Science, University of Nevada Reno, Reno, 1664 N Virginia St., Reno, NV 89557, USA; zaman@nevada.unr.edu

**Keywords:** spinal regeneration, mechanobiology, tissue engineering, neural scaffolds, bone repair, stem cell therapy, intervertebral disc degeneration, inflammation modulation, bioactive biomaterials, regenerative medicine

## Abstract

Spinal health depends on the dynamic interplay between mechanical forces, biochemical signaling, and cellular behavior. This review explores how key molecular pathways, including integrin, yeas-associated protein (YAP) and transcriptional coactivator with PDZ-binding motif (TAZ), Piezo, and Wingless/Integrated (Wnt) with β-catenin, actively shape the structural and functional integrity of spinal tissues. These signaling mechanisms respond to physical cues and interact with inflammatory mediators such as interleukin-1 beta (IL-1β), interleukin-6 (IL-6), and tumor necrosis factor alpha (TNF-α), driving changes that lead to disc degeneration, vertebral fractures, spinal cord injury, and ligament failure. New research is emerging that shows scaffold designs that can directly harness these pathways. Further, new stem cell-based therapies have been shown to promote disc regeneration through targeted differentiation and paracrine signaling. Interestingly, many novel bone and ligament scaffolds are modulating anti-inflammatory signals to enhance tissue repair and integration, as well as prevent scaffold degradation. Neural scaffolds are also arising. These mimic spinal biomechanics and activate Piezo signaling to guide axonal growth and restore motor function. Scientists have begun combining these biological platforms with brain–computer interface technology to restore movement and sensory feedback in patients with severe spinal damage. Although this technology is not fully clinically ready, this field is advancing rapidly. As implantable technology can now mimic physiological processes, molecular signaling, biomechanical design, and neurotechnology opens new possibilities for restoring spinal function and improving the quality of life for individuals with spinal disorders.

## 1. Introduction

**Figure 1:**
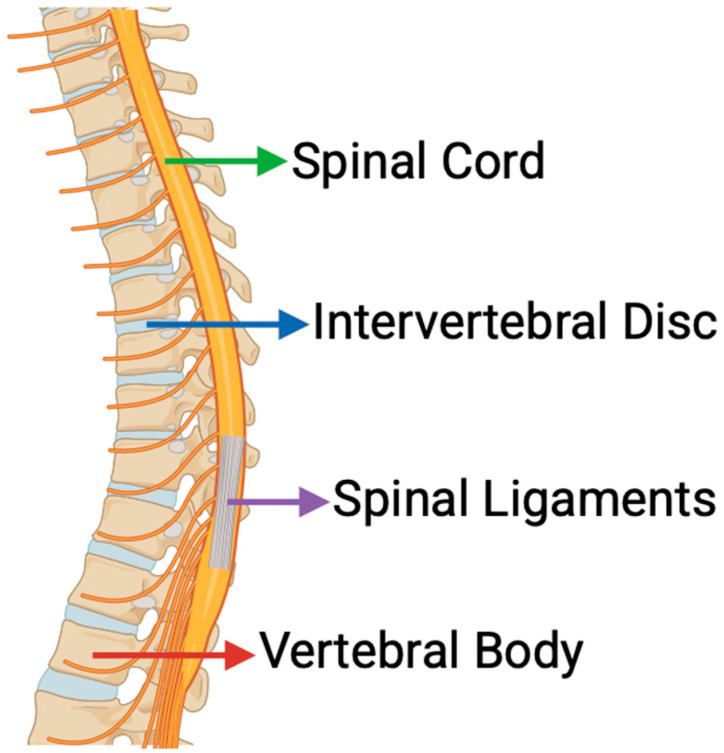
Key Anatomical Structures of the Spine Referenced Throughout This Review. Image adapted from www.biorender.com.

The human spine is the central pillar of structural support, mobility, and neural protection. Its health hinges on intricate molecular signaling pathways that orchestrate cellular responses to mechanical forces, inflammation, and tissue remodeling (Figure 1). These pathways are deeply rooted in immune modulation, cascade regulation, molecular machinery, and degenerative factors such as osteoarthritis, disc degeneration, and spinal cord injuries. As such, this review synthesizes the biochemical pathways behind the fundamental mechanobiology of the spine. Additionally, this review explores new computational approaches that are helping the physical structure of the spine, as well as helping clinicians and researchers understand what health factors go into spine maintenance and regeneration. The goal in doing so is to provide an updated review that also demonstrates how emerging technology and techniques are changing the landscape of spine health research.

This review is organized around the core anatomical structures of the spine. Section 2 looks at how mechanical signals are processed in the intervertebral discs (IVDs), vertebral bone, spinal cord, and ligaments. Section 3 shifts focus to inflammation and its role in these same tissues, covering major pathways like IL-6/Janus kinase/signal transducer and activator of transcription (JAK/STAT), TNF-α/nuclear factor kappa-light-chain-enhancer of activated B cells (NF-κB), and IL-1β/mitogen-activated protein kinase (MAPK). Section 4 takes a closer look at extracellular matrix remodeling, particularly through transforming growth factor-beta (TGF-β)/Smad signaling and matrix metalloproteinase (MMP) activity. Section 5 covers a range of regenerative approaches—from stem cell therapies and scaffold design to brain–computer interface technologies. Figure 1 provides a simple anatomical reference for the spinal regions discussed, which are revisited throughout the review in the context of how mechanobiological processes affect each one.

This review aims to connect mechanobiological signaling, inflammatory cascades, and regenerative technologies in the context of spinal health. By linking these molecular mechanisms to emerging tools in biomaterial design, stem cell therapies, and computational modeling, this review offers a comprehensive look at how basic science is driving new translational strategies. The goal of this review is to provide a cohesive framework that reflects both the complexity of spinal pathophysiology and the growing potential for integrated, cross-disciplinary interventions.

## 2. Methodology

This review was conducted between 15 January 2024 and 20 March 2025, following established guidelines for narrative reviews in biomedical engineering and regenerative medicine. The objective was to synthesize recent advances in mechanobiological signaling and regenerative strategies for spinal health by integrating molecular, biomechanical, and translational perspectives.

### 2.1. Literature Search Strategy

A structured search was performed across PubMed, Web of Science, Scopus, and Embase databases from January 2010 through March 2025, using keyword combinations such as “spinal mechanobiology,” “integrin-FAK signaling,” “Piezo channels,” “Wnt/β-catenin spine,” “stem cell therapies for intervertebral disc degeneration,” “inflammation modulation,” and “brain–computer interfaces in spinal cord injury.” Reference lists of relevant articles and recent review publications were manually screened to capture additional studies.

Inclusion criteria focused on:

- Original research articles, systematic reviews, and meta-analyses published in peer-reviewed journals.
- Experimental or computational studies elucidating molecular signaling pathways relevant to spinal tissues.
- Preclinical and clinical investigations on regenerative scaffolds, stem cell therapies, or neurotechnological interventions linked to spinal health.

Exclusion criteria included non-English publications, editorials, opinion pieces lacking primary data, and studies without mechanistic or translational relevance.

### 2.2. Selection and Data Extraction

All retrieved records were independently screened by two authors (R.K. and K.S.) between 1 February and 5 March 2024, with discrepancies resolved by consensus or a third reviewer (A.T.). Data were extracted on:

- Signaling pathways implicated in spinal tissue homeostasis (e.g., integrin-FAK-MAPK, YAP/TAZ, Piezo ion channels, Wnt/β-catenin, IL-6/JAK/STAT, TNF-α/NF-κB, TGF-β/Smad, and MMP-mediated ECM remodeling).
- Experimental models and intervention modalities (bioreactors, mechanical loading systems, scaffold engineering techniques, stem cell conditioning protocols).
- Translational approaches, including clinical trials and emerging brain–computer interface technologies (Neuralink reports up to June 2025).

Articles were categorized by spinal region (intervertebral discs, vertebrae, ligaments, spinal cord) and mechanobiological process (mechanotransduction, inflammation, ECM remodeling, regeneration). Findings were summarized and mapped onto a tissue-specific framework to facilitate cross-comparison of signaling mechanisms and therapeutic implications.

### 2.3. Quality Assessment

Each included study was evaluated for methodological rigor, reproducibility of findings, and translational relevance using modified criteria adapted from PRISMA guidelines and the NIH quality assessment tool for preclinical research. In vitro studies were assessed for cell source heterogeneity, biomechanical loading fidelity, and quantification of pathway activation. Animal studies were assessed for species model relevance, loading parameters, and durability of regenerative outcomes. Clinical trials were evaluated for study design, cohort size, and long-term outcome reporting.

### 2.4. Synthesis of Evidence

Findings were integrated into a multi-layered conceptual model linking mechanical stimuli, inflammatory mediators, and regenerative pathways. Figures (generated via BioRender) were constructed between February and April 2025 to visualize interconnections among pathways and therapeutic strategies. This structured approach enabled the identification of emerging consensus mechanisms, critical knowledge gaps, and future directions in spine health research.

## 3. Mechanotransduction in Spinal Tissues

**Figure 2:**
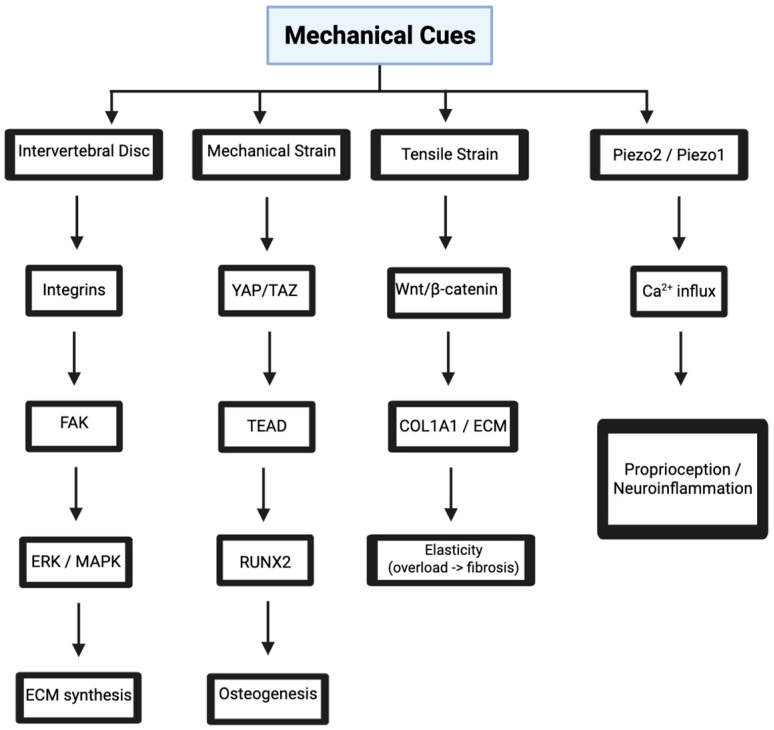
Mechanical cues regulate cell behavior in spinal tissues through key signaling pathways. Integrin and YAP/TAZ activation promote ECM synthesis and osteogenesis via FAK–ERK/MAPK and TEAD–RUNX2 signaling. Tensile strain activates Wnt/β-catenin, influencing ECM remodeling, while Piezo-mediated calcium influx drives proprioception and neuroinflammation. Image adapted from www.biorender.com.

Mechanotransduction is a process where cells convert mechanical stimuli into biochemical signals to respond or initiate movement [1]. The spine’s intervertebral discs, vertebrae, ligaments, and cord constantly experience this mechanical loading from posture, movement, and weight-bearing. Even without movement, weight-bearing alone triggers molecular pathways that regulate cellular behavior, extracellular matrix (ECM) maintenance, and inflammatory responses [1,2]. Thus, understanding mechanotransduction in the spine is critical for developing tissue-engineered solutions for conditions where mechanical imbalances disrupt signaling homeostasis, namely, degenerative disc disease and osteoarthritis (Figure 2).

### 3.1. Integrin-Mediated Signaling in Intervertebral Discs

IVDs cushion the spine and absorb compressive forces during movement [3]. Integrins, transmembrane receptors, mediate mechanotransduction by linking the ECM to the cytoskeleton [4]. In the nucleus pulposus (NP), integrins such as α5β1 bind to fibronectin and transmit mechanical signals that activate focal adhesion kinase (FAK) [5]. FAK will then phosphorylate downstream targets, initiating pathways like extracellular signal-regulated kinase (ERK)/MAPK, which regulate gene expression for ECM components such as collagen II and aggrecan [6]. These molecules will then maintain disc hydration and fundamental resilience to absorb shock from weight bearing. Normally, this repetitive process ensures that movements can be quick and painless. If patients excessively weight-bear or the ECM degrades, this will largely dysregulate the integrin signaling process. In turn, production of a proteoglycan, called aggrecan, that makes up the cartilage structure, will drop, and this will accelerate disc degeneration [7]. Integrin signaling is sensitive to mechanical inputs, particularly cyclic loading, which refers to the repetitive application of mechanical force over time (e.g., during walking, lifting, or bending) [8]. Moderate mechanical stress enhances integrin activation and promotes anabolic gene expression [9]. However, prolonged or excessive compression disrupts integrin-ECM interactions, triggering catabolic pathways [10]. For instance, high loads increase MMP expression, which are zinc-binding proteolytic enzymes that can remodel the ECM, and this in turn degrades collagen and proteoglycans [9,10]. This imbalance ultimately leads to disc herniation. Emerging engineering strategies such as scaffolds with tunable stiffness are especially beneficial here because they can match the stiffness of surrounding tissue. Specifically, these scaffolds optimize osteointegration by enhancing osteoblast mechanosensitivity within a narrow stiffness window (~0.7–3 megapascals (MPa)), promoting maximal f-actin polymerization and mineral deposition at the bone-implant interface, thereby reducing stress shielding and improving long-term implant fixation [11,12,13].

Integrin signaling also intersects with inflammatory pathways in the IVD [14]. Mechanical overload induces cytokine release, such as IL-6 (a key inflammatory mediator), which amplifies MMP activity. IL-6 then binds to its receptor, activating JAK/STAT signaling [15,16]. This then upregulates catabolic genes. This feedback loop is what exacerbates disc degeneration. The clinical relevance of integrin signaling extends to regenerative medicine as well. Stem cell therapies for IVD repair rely on mechanotransductive cues to direct differentiation. Mesenchymal stem cells (MSCs) cultured on stiff scaffolds upregulate integrin expression [17], enhancing chondrogenic differentiation [18]. This process mirrors native disc cell behavior, where mechanical cues drive ECM synthesis [1,2,3]. However, translating these findings to clinical practice requires scaffolds that replicate the disc’s viscoelastic properties so that integrins are not over-activated. Excessively stiff substrates risk inducing aberrant mechanotransduction, leading to cytoskeletal hyperactivation and premature matrix degradation via upregulated catabolic signaling. Additionally, many current scaffold systems lack the microelastic gradients required to guide region-specific differentiation in the IVD niche [19]. Novel biomaterials, such as water-annealed glutenin-chitosan composites, show promise in addressing this limitation by offering tunable stiffness and microarchitectural control [20], yet their translation to orthopedic applications remains underexplored.

### 3.2. YAP/TAZ Pathway in Vertebral Bone Mechanobiology

#### 3.2.1. YAP/TAZ in Bone

Vertebral bones withstand extensive compressive and tensile forces to maintain spinal stability. The YAP/TAZ pathway is a mechanosensitive transcriptional regulator that governs osteoblast activity in response to these forces [21]. Mechanical loading activates YAP/TAZ by inhibiting their phosphorylation, which facilitates nuclear translocation. In the nucleus, YAP/TAZ bind TEA domain (TEAD) transcription factors and upregulate osteogenesis-promoting genes such as Runt-related transcription factor 2 (RUNX2) [22,23]. Normally, this cycle will balance bone remodeling with mechanical demands. Therefore, YAP/TAZ is very important for maintaining the vertebrae.

#### 3.2.2. YAP/TAZ and Inflammation

Osteoporosis can arise due to significantly reduced mechanical loading (e.g., through a lack of mobility or inactive lifestyle), and it enhances YAP/TAZ phosphorylation [21,22,23]. This makes sense as phosphorylation would inactivate a less frequently used mechanism and sequester it in the cytoplasm. In turn, osteoblasts cannot differentiate, and bone loss begins. Unfortunately, this may be a reinforcing cycle as vertebral fractures can become more common and hinder mobility even further. YAP/TAZ also modulates inflammatory responses in the vertebral bone. Mechanical stress induces IL-1β release, which suppresses YAP/TAZ activity through NF-κB signaling. This inflammatory cascade reduces osteoblast viability and accelerates bone resorption [24]. Osteoarthritic patients often experience sclerosis in their vertebral endplates because of IL-1β-driven inflammation exacerbating YAP/TAZ dysregulation. This interplay between YAP/TAZ and mechanotransduction can inform new regenerative strategies. For example, in tendon fibroblasts, 4% uniaxial stretching at 0.5 Hertz (Hz) attenuated IL-1β-induced cyclooxygenase-2 (COX-2) and MMP-1 expression and reduced prostaglandin E2 (PGE2) secretion [25], highlighting the anti-inflammatory potential of sub-physiological strain. Analogously, graded spinal loading in osteoporotic patients may downregulate NF-κB-mediated YAP/TAZ suppression, promoting osteoblast viability while curbing catabolic remodeling [26]. These findings support the development of biomechanically tuned rehab regimens and viscoelastic scaffolds engineered to deliver anti-inflammatory mechanotransductive cues within the degenerating vertebral niche.

### 3.3. Piezo Channels in Neural Mechanosensing

#### 3.3.1. Piezo Signaling in Injury

The spinal cord relies on mechanosensitive ion channels like Piezo1 and Piezo2 to detect stimuli [27]. Piezo channels are specifically activated by membrane tension and regulate calcium influx in neurons and glia. In the spinal cord, Piezo2 in sensory neurons mediates proprioception. Dysfunctional Piezo signaling therefore disrupts core spinal function [28]. In spinal cord injuries (SCIs), mechanical trauma damages neurons, impairing Piezo2. This disrupts proprioceptive feedback and induces motor deficits. On the other hand, Piezo1 is expressed in astrocytes and modulates inflammatory responses post-injury [29]. Excessive mechanical stress activates Piezo1, then releases IL-6 and TNF-α, which drive up neuroinflammation [30]. Piezo channels also influence spinal cord regeneration. Many theoretical and animal models have also emerged around how Piezo signaling can be manipulated for regeneration. In zebrafish, Piezo1 activation enhances axonal growth, suggesting a conserved role in neural repair [31]. While Piezo1 is predominantly expressed by astrocytes in the mammalian spinal cord, recent studies have also demonstrated its upregulation in neurons following spinal injury. In particular, LOX-driven ECM remodeling has been shown to induce neuronal Piezo1 expression, intensifying calcium influx and ferroptosis in injury models [32]. Inhibition of Piezo1 with agents like GsMTx4 not only attenuates neuronal damage but also restores cognitive function in hypoxia-induced brain injury [16,33]. These findings suggest that Piezo1 may contribute to spinal cord regeneration and neuroinflammation through both astrocytic signaling and neuron-intrinsic pathways.

#### 3.3.2. Piezo in Chronic Pain

Piezo channel regulation may also help with chronic pain management. Aberrant Piezo2 activation in dorsal root ganglia contributes to mechanical allodynia [34]. Recent preclinical models demonstrate that targeted depletion or chemogenetic silencing of Piezo2-expressing nociceptors effectively blocks mechanical allodynia and weight-bearing pain without exacerbating joint degeneration [35,36,37,38]. Co-expression of Piezo2 and TrkA in human DRGs underscores a conserved NGF-Piezo2 signaling axis implicated in chronic osteoarthritis pain [39]. These findings have catalyzed new developments of intra-articular Piezo2 inhibitors as proprioception-sparing analgesics [38]. In parallel, emerging chemogenetic approaches, including DREADD-based neuromodulation, are being explored for selective targeting of Piezo2-expressing nociceptors [35].

### 3.4. Wnt/β-Catenin Signaling in Ligament Mechanobiology

#### 3.4.1. Role in Ligament Homeostasis, Fibrosis, and Inflammatory Modulation

Spinal ligaments, like the ligamentum flavum, stabilize the spine under tensile forces. Wnt/β-catenin signaling is a mechanosensitive pathway that regulates ligament fibroblast activity by activating Wnt ligands, which bind Frizzled receptors and stabilize β-catenin [40]. Nuclear β-catenin upregulates genes like Collagen Type I Alpha 1 Chain (COL1A1), encoding collagen I, and ultimately strengthens the ligament [41]. This pathway maintains ligament elasticity, preventing hypermobility and spinal instability. In healthy spines, Wnt/β-catenin balances ECM synthesis with degradation, supporting dynamic movement [42,43,44]. Constant loading disrupts Wnt/β-catenin signaling, compromising ligament function [45]. In hypertrophic ligamentum flavum, excessive tensile stress overactivates β-catenin, increasing collagen deposition and fibrosis. This stiffens ligaments, contributing to spinal stenosis [46]. Pharmacological Wnt inhibitors, such as sclerostin, could mitigate fibrosis, restoring ligament flexibility [47]. New tissue-engineered ligaments are beginning to arise that can deliver controlled mechanical stimuli, which would normalize Wnt/β-catenin activity and hypertrophy [48,49,50]. Inflammatory mediators modulate Wnt/β-catenin signaling in ligaments. IL-6, induced by mechanical overload, suppresses Wnt activity via Suppressor of Cytokine Signaling 3 (SOCS3), reducing collagen synthesis [51]. This imbalance weakens ligaments, increasing injury risk.

#### 3.4.2. Wnt-Guided Ligament Regeneration Strategies

Fortunately, Wnt/β-catenin signaling informs regenerative approaches for ligament injuries. MSCs cultured under tensile strain upregulate β-catenin and have been shown to enhance tenogenic differentiation [52,53,54]. Scaffolds that replicate ligament mechanics could direct MSC fate, producing functional tissue replacements [55]. Emerging scaffold designs now leverage biomechanical stimuli to spatially modulate Wnt/β-catenin gradients and tenogenic gene expression in situ [56]. Recent ligament-on-a-chip platforms and high-fidelity microphysiological systems (MPS) incorporating tensile loading have validated β-catenin-mediated tenogenesis in human MSCs under physiomimetic strain [52,57,58,59]. These models are accelerating the translation of mechanoresponsive, stem cell-laden scaffolds for enthesis repair and next-generation ligament reconstruction.

## 4. Inflammatory Signaling in Spinal Health

**Figure 3:**
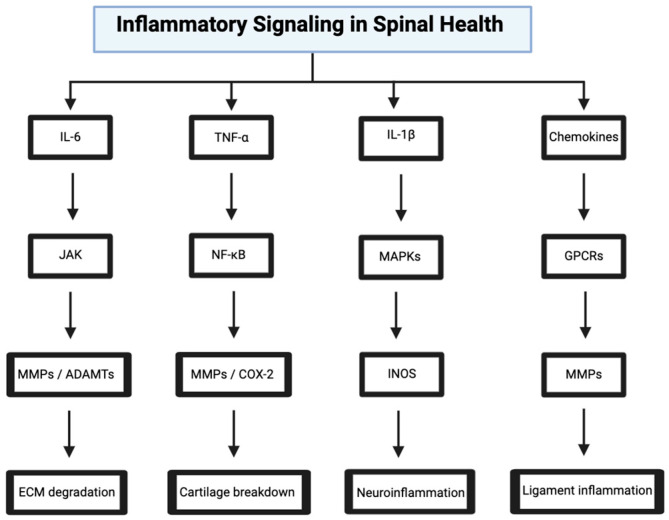
Inflammatory signaling pathways contributing to spinal degeneration. Key cytokines—including IL-6, TNF-α, IL-1β, and chemokines—trigger downstream cascades via JAK/STAT, NF-κB, MAPK, and GPCR pathways, promoting ECM degradation, cartilage breakdown, neuroinflammation, and ligament inflammation. Image adapted from www.biorender.com.

Inflammation can both mediate repair and exacerbate degeneration. Inflammatory signaling is driven by cytokines like IL-6, TNF-α, and IL-1β in response to mechanical stress, injury, and infection [1,2,15,18,25]. Dysregulated inflammation can also be linked to many spinal disorders and can even be improperly suppressed through post-operative medication (Figure 3).

### 4.1. IL-6/JAK/STAT Pathway in Disc Degeneration

As previously mentioned, IL-6 is a pleiotropic cytokine that drives inflammatory responses in IVDs. Mechanical overload or injury triggers IL-6 release from NP cells, and the JAK/STAT pathway activates [14,15,16]. IL-6 binds its receptor, recruiting JAK kinases that then phosphorylate Signal Transducer and Activator of Transcription 3 (STAT3). Nuclear STAT3 upregulates MMPs and A Disintegrin And Metalloproteinase with Thrombospondin Motifs (ADAMTS) enzymes, degrading ECM components like aggrecan [60,61]. This catabolic cascade reduces disc height and causes pain. Chronic IL-6 signaling perpetuates inflammation in the IVD. STAT3 also induces IL-6 expression, creating a positive feedback loop [62]. This amplifies neuroinflammation, sensitizing nociceptors and contributing to chronic back pain. However, anti-IL-6 treatments such as tocilizumab interrupt this cycle and reduce MMP activity and pain [63]. Additionally, newly studied IVD scaffolds integrate high-molecular-weight hyaluronic acid (HMWHA) and genipin-crosslinked fibrin to suppress IL-6 and TNF-α signaling through CD44 and NF-κB inhibition, respectively, while preserving ECM architecture [64]. Injectable hydrogels loaded with lactate oxidase-catalase fusion enzymes have been shown to modulate acidic, reactive oxygen species-rich NP microenvironments, reversing immune cell recruitment and IL-6 amplification [65,66,67]. Ideally, this can be a part of post-operative recovery and prevent over-inflammation through the JAK/STAT pathway. Next-generation nanoparticle-based delivery platforms, including poly(lactic-co-glycolic acid) (PLGA) or polycaprolactone (PCL) nanocarriers encapsulating anti-IL-6 small interfering RNA (siRNA) or interleukin-1 receptor antagonist (IL-1ra) peptides, can also sustain release kinetics and target cytokine blockade in avascular disc tissue [68], which can be helpful in rehabilitation.

### 4.2. TNF-α/NF-κB Pathway in Osteoarthritis

#### 4.2.1. Mechanotransduction and Catabolic Signaling

TNF-α is another pro-inflammatory cytokine connected to osteoarthritis [69]. Mechanical stress or injury induces TNF-α release from chondrocytes, activating NF-κB. TNF-α then binds tumor necrosis factor receptor 1 (TNFR1), recruiting TNF receptor-associated factor 2 (TRAF2), which activates inhibitor of kappa B kinase (IKK) and phosphorylates inhibitor of kappa B (IκB) [70,71]. This releases NF-κB, which will translocate to the nucleus and upregulate MMPs and COX-2. These enzymes subsequently degrade cartilage and induce pain, contributing to osteoarthritis [72]. Excessive TNF-α signaling will significantly accelerate cartilage loss, and NF-κB also induces IL-1β, which amplifies inflammation. This creates a catabolic environment and erodes endplates [73].

#### 4.2.2. Anti-TNF Therapies and Scaffold-Based Modulation

Anti-TNF-α therapies such as adalimumab can reduce NF-κB activity and therefore slow degradation [74]. However, new tissue-engineered cartilage seems promising. Bioengineered constructs that use ECM scaffolds and are derived from collagen, hyaluronic acid, or silk fibroin seem to be able to modulate multiple inflammatory signaling pathways [75,76,77]. This includes NF-κB, p38 MAPK, and c-Jun N-terminal kinase (JNK), and they subsequently reduce TNF-α-induced catabolic mediators, including MMP-13, COX-2, and ADAMTS-5 [77]. These scaffolds are further enhanced through growth factor loading with transforming growth factor beta 1 (TGF-β1), insulin-like growth factor I (IGF-I), or fibroblast growth factor 2 (FGF-2), which not only stimulate anabolic processes such as type II collagen and aggrecan synthesis but also suppress IL-1β and TNF-α-driven transcriptional programs by downregulating IKKβ phosphorylation and restoring IκBα stability [78,79,80,81].

#### 4.2.3. Immunomodulatory Stem Cell Strategies

In parallel, preconditioning of mesenchymal stem cells (MSCs) with decellularized stem cell matrices (DSCMs) has been shown to be a useful immunomodulatory approach [82]. These DSCM-expanded MSCs show enhanced resilience against IL-1β- and TNF-α-induced inflammatory cues, with upregulation of antioxidant defenses and increased secretion of anti-inflammatory cytokines like IL-10 [82]. Mechanistically, DSCM preconditioning enhances MSC expression of chondrogenic transcription factors while simultaneously downregulating NF-κB nuclear translocation and pro-apoptotic gene expression [83]. Notably, intra-articular delivery of DSCM-preconditioned MSCs in large-animal models results in robust hyaline-like cartilage regeneration, reduced type I collagen deposition, and minimal macrophage infiltration [84].

### 4.3. IL-1β/MAPK Pathway in Spinal Cord Injury

#### 4.3.1. Neuroinflammatory Cascade and Biomaterial-Based Modulation

IL-1β is another potent inflammatory cytokine that shapes the spinal cord’s response to injury. Trauma induces IL-1β release from microglia, activating MAPK pathways (p38, JNK, ERK) [85,86]. IL-1β then binds IL-1R, recruiting MyD88, which activates TAK1. TAK1 will then phosphorylate MAPKs. Nuclear MAPKs upregulate pro-inflammatory genes, including IL-6 and TNF-α, therein exacerbating neuroinflammation [86,87]. This cascade impairs neuronal survival and contributes to many motor deficits in SCIs. Thus, chronic IL-1β signaling perpetuates neuroinflammation in SCI patients. MAPKs also induce iNOS, producing nitric oxide, which damages neurons. This creates a toxic microenvironment that further hinders regeneration [88]. Fortunately, new tools are being designed to directly attenuate TNF-α–driven inflammatory cascades and catabolic matrix remodeling. ECM-mimetic scaffolds constructed from high-density type II collagen, gelatin-methacrylate (GelMA), or hyaluronic acid-based hydrogels have been functionalized with anti-inflammatory agents such as TGF-β3, IL-10, or FGF-18, enabling localized inhibition of the NF-κB and MAPK pathways in chondrocytes and synovial fibroblasts [89,90]. These scaffolds suppress the expression of downstream targets, including MMP-13, ADAMTS-5, iNOS, and COX-2 by interfering with TRAF signaling and stabilizing cytoplasmic IκBα, thus preventing nuclear translocation of p65/p50 NF-κB dimers [89,90,91]. Some other strategies found in the literature are nanoparticle-based delivery systems (specifically, PLGA or mesoporous silica nanoparticles) to encapsulate small molecule IKKβ inhibitors or anti-TNF-α siRNA [91,92,93,94] and spatiotemporally release within the hypoxic cartilage environment.

#### 4.3.2. MSC Preconditioning for Immunomodulation

Saparov et al. (2016) showed that preconditioning mesenchymal stem cells (MSCs) using hypoxic three-dimensional culture or inflammatory cytokines such as IL-1β and TNF-α enhances their immunomodulatory capacity [95]. These preconditioned MSCs increase anti-inflammatory mediator expression, specifically the IL-1 receptor antagonist PGE-2, as well as heme oxygenase-1 (HMOX1), while reducing activation of pro-inflammatory pathways, including NF-κB and MAPK. They also exhibit greater resistance to apoptosis and upregulate survival genes such as BCL-2 and AKT. In addition, they promote chondrogenic activity by increasing the expression of SOX9 and aggrecan. In animal models of inflammatory arthritis, intra-articular injection of these MSCs significantly reduces synovial thickening, subchondral erosion, and local TNF-α levels, while restoring cartilage with hyaline-like features and improved biomechanical strength [96]. Thus, Saparov et al. suggest that by attenuating these inflammatory cascades, MSCs may preserve extracellular matrix integrity, suppress catabolic signaling, and support functional recovery in damaged tissue.

### 4.4. Chemokine Signaling in Ligament Inflammation

Chemokines such as CCL2 and CXCL8 also regulate inflammatory responses in spinal ligaments [96]. Mechanical strain or injury induces chemokine release from fibroblasts, activating G-protein-coupled receptors (GPCRs). CCL2 binds CCR2, recruiting monocytes, which release IL-6 and TNF-α [97,98]. This amplifies inflammation, contributing to ligament hypertrophy and spinal stenosis. CXCL8, by activating CXCR1/2, enhances neutrophil infiltration, exacerbating tissue damage [98]. Chronic chemokine signaling will therefore perpetuate ligament inflammation. CCR2 activation induces NF-κB, upregulating MMPs, which degrade collagen. This weakens ligaments and increases injury risk. While anti-CCL2 antibodies could reduce monocyte recruitment [99], emerging research is also showing that it can be beneficial to directly target TNF-α and NF-κB signaling at the cellular and molecular levels. DSCM expands MSCs with enhanced resistance to IL-1β and TNF-α, maintaining viability and suppressing inflammatory cascades through reduced NF-κB nuclear translocation [100]. These MSCs show increased expression of IL-1Ra, HMOX1, and SOD2, and decreased TNFR1 and pro-apoptotic genes, enhancing chondrogenic potential [100,101,102]. Intra-articular delivery of DSCM-expanded MSCs in TNF-α-induced arthritis models reduces synovial thickness, inflammatory cytokines, and ECM degradation [103]. Additionally, nanocarriers incorporating anti-TNF-α siRNA or small-molecule IKKβ inhibitors have proven that they can maintain efficacy in hypoxic, acidic environments [104]. These systems block TRAF6-IKKβ-IκBα signaling, reduce MMP13 and ADAMTS5 expression, and restore anabolic markers like ACAN and COL2A1. Together, these approaches offer multi-tiered suppression of catabolic signaling, promote immune homeostasis, and support durable cartilage regeneration.

It is also important to note that chemokines intersect with mechanotransduction in ligaments [105]. Tensile strain activates integrins, thereby enhancing CCL2 production via FAK. This proto-inflammatory pathway then amplifies GPCR signaling [106]. Biomaterials that mimic physiological tension could theoretically normalize integrin activation and reduce chemokine levels. Clinically, chemokine signaling is also a significant biomarker [107,108,109], such as how elevated CCL2 can correlate with stenosis symptom severity.

## 5. ECM Remodeling and Spinal Stability

**Figure 4:**
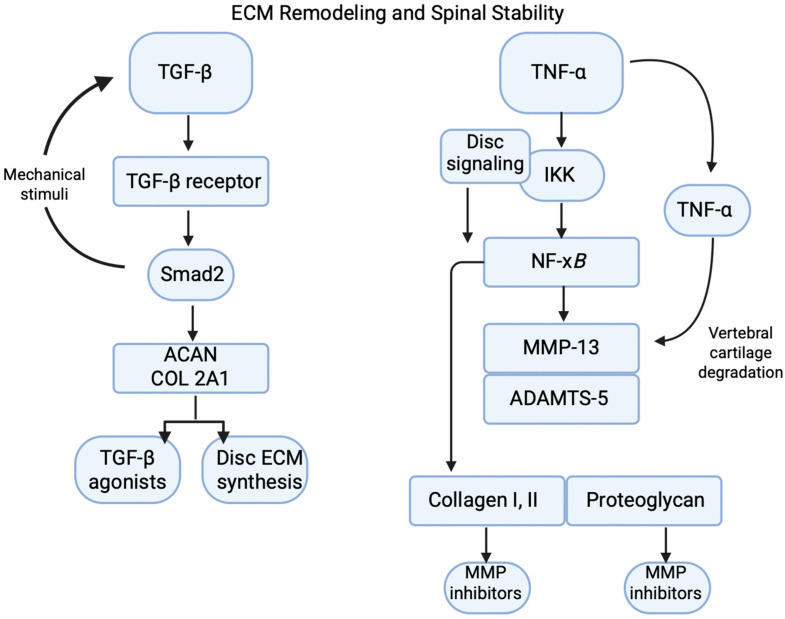
TGF-β and TNF-α mediate opposing pathways in the extracellular matrix (ECM) remodeling of the intervertebral disc, as discussed in Section 4. TGF-β signaling promotes ECM synthesis through Smad2-dependent activation of aggrecan (ACAN) and type II collagen (COL2A1), while TNF-α activates NF-κB signaling to upregulate MMP-13 and ADAMTS-5, driving proteolytic degradation and loss of disc structural integrity. Image adapted from www.biorender.com.

The ECM must constantly modulate between synthesis and degradation. Molecular signaling pathways, including TGF-β, MMPs, and TIMPs, regulate ECM remodeling in spinal tissues [110]. These pathways respond to mechanical cues and inflammation, gradually shaping tissue architecture. When ECM remodeling goes wrong, many disorders, including ligament fibrosis, disc degeneration, and osteoarthritis, can arise. Understanding these pathways is therefore essential for developing tissue-engineered solutions for affected patients (Figure 4).

### 5.1. TGF-β/Smad Pathway in Disc ECM Synthesis

TGF-β is a growth factor that drives ECM synthesis in IVDs. When the spine is stimulated, TGF-β is released from NP cells and will activate Smad signaling. TGF-β binds to its receptor, phosphorylating Smad2/3, which complexes with Smad4 and translocates to the nucleus. Smad2 upregulates genes like ACAN and COL2A1, encoding aggrecan and collagen II, respectively [111]. These proteins are overall responsible for maintaining disc hydration and resilience, supporting spinal mobility. In aged discs, reduced mechanical responsiveness decreases TGF-β expression, which means Smad activity is downregulated. This reduces aggrecan synthesis and compromises disc hydration. There are TGF-β agonists, including recombinant proteins, that could restore Smad signaling and help repair the ECM [112].

Recent work has identified DSCM as a potent immunomodulatory platform for expanding MSCs with enhanced resistance to TNF-α and IL-1β signaling. DSCM-expanded MSCs display reduced TNFR1 expression, decreased NF-κB nuclear translocation, and lower expression of pro-apoptotic genes, while upregulating IL-1Ra, HMOX1, and SOD2 [113,114]. These cells maintain chondrogenic gene expression, including SOX9 and COL2A1, even under inflammatory challenge. Intra-articular injection of DSCM-MSCs in TNF-α-induced arthritis models reduces synovial inflammation, suppresses expression of MMP13 and ADAMTS5, and preserves cartilage integrity [115,116]. Parallel studies have developed nanocarriers such as PLGA and mesoporous silica nanoparticles to deliver anti-TNF-α siRNA or IKKβ inhibitors directly to inflamed joints [117,118,119]. These systems inhibit TRAF6-mediated NF-κB activation, restore IκBα stability, and maintain anabolic signaling in chondrocytes. Collectively, these findings suggest that combining immunomodulatory MSCs with targeted cytokine interference offers a robust strategy to counteract TNF-α-driven joint degeneration and promote sustained tissue regeneration.

### 5.2. MMP-13/TNF-α Pathway in Vertebral Cartilage Degradation

MMP-13 is a collagenase that drives ECM degradation in vertebral endplates [120]. Mechanical stress induces TNF-α, which upregulates MMP-13 through NF-κB. TNF-α binds to TNFR1, activating IKK, which phosphorylates IκB and releases NF-κB. Nuclear NF-κB upregulates MMP-13, and collagen I and II are degraded. This erodes cartilage and thus drives osteoarthritis [121]. NF-κB also induces ADAMTS-5, and proteoglycan will be lost if this pathway is overactive [122]. This creates a catabolic microenvironment, causing endplate sclerosis and pain. MMP inhibitors, such as doxycycline, reduce ECM degradation and have been shown to effectively slow osteoarthritis progression [122]. Recent research demonstrates that Amygdalin (AMD), a bioactive cyanogenic glycoside, significantly attenuates NF-κB signaling by inhibiting p65 and IκBα phosphorylation in cartilage endplate chondrocytes [123]. In both in vivo LSI-induced IDD models and IL-1β-stimulated in vitro systems, AMD reduced MMP-13 and TNF-α expression while preserving type II collagen integrity [124]. This dual anti-catabolic and anti-inflammatory action restores endplate microarchitecture and disrupts the ECM-inflammation feedback loop. Such findings position AMD as a mechanistically targeted modulator of NF-κB signaling with translational potential for biotherapeutic intervention in discogenic osteoarthritis.

## 6. Regenerative Strategies for Spinal Health

**Figure 5:**
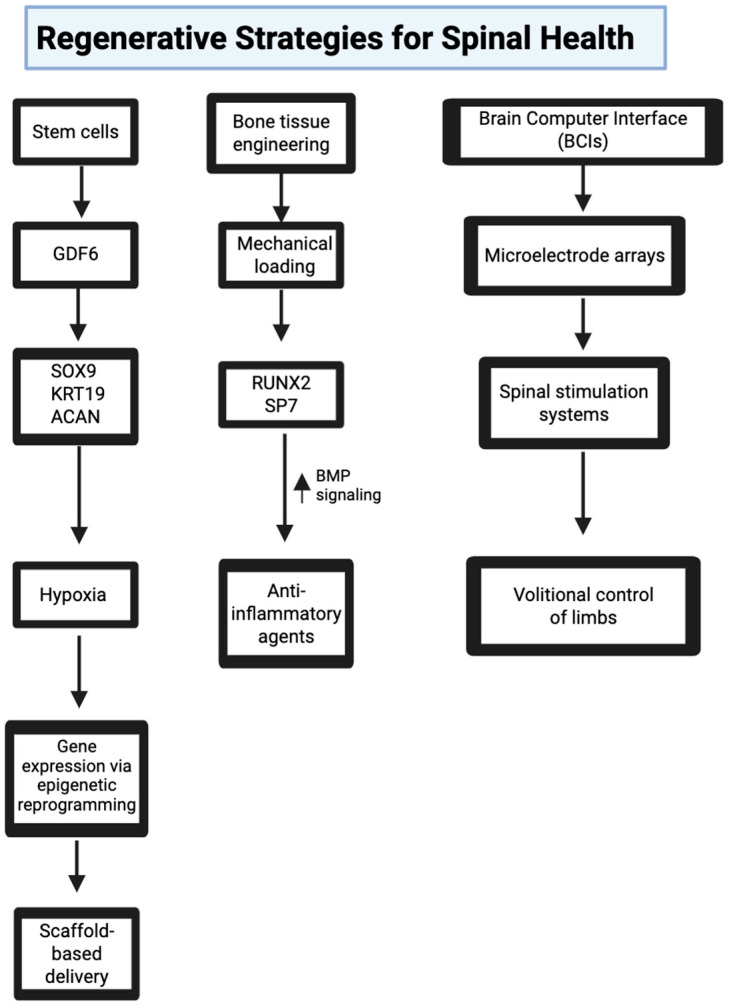
Regenerative strategies highlighted in Section 5 target different aspects of spinal health—ranging from stem cell therapies for disc repair, to bone tissue engineering using mechanical cues, to neural technologies like BCIs that support functional recovery. Each approach offers a unique angle for addressing degeneration and promoting long-term stability. Image adapted from www.biorender.com.

Molecular signaling fundamentally drives stem cell differentiation, tissue repair, and successful biomaterial integration. Thus, researchers developing regenerative medicine strategies must account for mechanical, inflammatory, and epigenetic cues to ensure that regenerative interventions are not only biologically compatible but also functionally durable across diverse pathologies (Figure 5).

### 6.1. Stem Cell Therapies for Disc Regeneration

Many new MSC therapy advancements are coming into mainstream research for intervertebral disc degeneration.

#### 6.1.1. Morphogen-Induced Discogenic Differentiation

Bone marrow-derived MSCs (BM-MSCs) and adipose-derived stem cells (ADSCs) are still the gold standard as cell sources due to their immunomodulatory profiles, hypoxia resilience, and NP-like differentiation [125,126]. Clarke et al. (2014) demonstrated that growth differentiation factor 6 (GDF6) promotes lineage-specific discogenic differentiation of BM-MSCs by upregulating NP markers (SOX9, KRT19, ACAN), enhancing the expression of ECM components, including aggrecan and collagen II [127]. These findings suggest that GDF6-driven discogenic differentiation enhances ECM production and phenotype stability. MSCs can then be potentially better equipped or, at the very least, more resistant to this hostile environment. Similarly, Wei et al. (2014) reported that MSCs implanted into the rabbit discs maintained long-term viability and synthesized key NP matrix proteins, leading to improved hydration and disc height [128]. These findings have catalyzed the use of GDF6, TGF-β3, and other morphogens in scaffold-based delivery platforms to promote phenotypic stability post-injection.

#### 6.1.2. Hypoxic Preconditioning and Epigenetic Modulation

Hypoxic preconditioning is another emerging strategy to boost MSC resilience. Yang et al. (2022) showed that hypoxia-preconditioned MSCs had reduced apoptosis, increased collagen II deposition, and greater aggrecan retention in rat models of IDD [129]. These findings are consistent with other researchers who observed that low oxygen tension enhances NP-like gene expression in ADSCs through epigenetic reprogramming and HIF-1α stabilization [130,131]. Epigenetic modifiers like histone deacetylase inhibitors are also now being integrated into hydrogel systems to further modulate gene expression and differentiation trajectories [132].

#### 6.1.3. Biomechanically Tuned Scaffolds and Bioreactor Platforms

Scaffold technologies have also evolved to accommodate the biomechanical demands of the IVD. Yang et al. (2024) demonstrated that viscoelastic sodium alginate-gelatine hydrogels, when seeded with BM-MSCs and subjected to dynamic compression, significantly enhanced TRPV4 activation and intracellular calcium influx, which in turn activated Wnt/β-catenin signaling and promoted osteogenic differentiation [133]. Additionally, composite scaffolds with fast stress relaxation properties facilitated MSC migration, improved adhesion, and upregulated key osteogenic markers such as RUNX2 and COL1A1 [133]. Dynamic bioreactor systems applying physiologic compressive strain (1.5%) were found to synergize with viscoelastic cues, maximizing osteogenesis through mechanical tuning of the TRPV4-Ca^2+^-β-catenin axis.

#### 6.1.4. Genetically Engineered MSCs and Extracellular Vesicles

Emerging studies have also begun experimenting with genetically engineered MSCs. Preclinical models are evaluating CRISPR-mediated silencing of senescence markers (e.g., p16INK4a) and overexpression of ECM-stabilizing proteins such as COMP and fibromodulin [134]. Additionally, extracellular vesicles (EVs) derived from MSCs—particularly exosomes carrying miR-140 and miR-21—are being explored as cell-free alternatives that replicate paracrine therapeutic effects while minimizing overcell viability and tumorigenicity [135]. Future directions in this avenue should include integrating MSCs into 3D-printed disc-like constructs, applying matrix-mimetic cues for zonal control of differentiation, and using ex vivo organ culture systems for high-throughput testing.

### 6.2. Bone Tissue Engineering for Vertebral Fractures

Currently, orthopedic surgeons will insert autologous bone grafts to treat complex bone defects. This involves harvesting bone from the patient’s own body (e.g., the iliac crest) and transplanting it to the fracture site [136,137]. However, many patients may not be able to withstand this harvest. Fortunately, tissue-engineered bone constructs are emerging as a potential solution for fracture fixation. Mechanical loading of osteoblast-seeded scaffolds activates nuclear translocation of YAP and TAZ, which in turn upregulate osteogenic transcription factors such as RUNX2 and SP7 that promote ECM deposition and mineralization [138]. Scaffolds engineered with high compressive strength and viscoelastic compliance mimic vertebral biomechanical properties to enable structural support and biological integration. In preclinical models, these constructs have successfully repaired vertebral defects, restored bone density, and improved load-bearing capacity.

Despite this progress, inflammation is still a major impediment. Trauma-induced IL-1β signaling activates the NF-κB pathway, suppressing YAP and TAZ activity and impairing osteoblast viability and differentiation [138]. To counter this, patients should also simultaneously receive anti-inflammatory agents such as TNF-α inhibitors or IL-1 receptor antagonists in the scaffold matrix. Scaffolds functionalized with bioactive peptides also stabilize BMP signaling and improve osteoinductive potential, particularly in osteoporotic contexts where systemic inflammation and matrix fragility are common [139,140,141]. Recent studies provide additional insight into inflammatory modulation. Zeng et al. (2024) demonstrated that Amygdalin (AMD) inhibits NF-κB signaling by preventing IκBα and p65 phosphorylation, thereby reducing downstream expression of MMP-13 and ADAMTS-5 [123]. In rat LSI-induced disc degeneration models, AMD preserved type II collagen and proteoglycan content while reducing TNF-α and IL-6 secretion [123,142]. Epigenetic modulation complements these regenerative efforts. Mechanical loading reduces repressive histone marks (e.g., H3K27me3) while increasing permissive modifications (e.g., H3K9ac) at osteogenic gene promoters [143], thereby enhancing transcriptional output.

However, the immunogenicity of bioengineered scaffolds remains a key consideration. Even when designed to be biocompatible, some synthetic or decellularized materials can still trigger inflammation, whether due to leftover cellular debris, certain cross-linking chemicals, or how the scaffold surface interacts with surrounding tissue. These responses can interfere with proper bone integration or lead to unwanted fibrous tissue formation. Strategies such as adding anti-inflammatory agents or modifying the scaffold surface with hydrophilic coatings are being explored to reduce these risks and support long-term success [140].

### 6.3. Neural Technology for Rehabilitation and Support

Recent breakthroughs in neuroengineering have also dramatically expanded the therapeutic landscape. As is the case with many CNS-related conditions, complementary treatments alongside primary treatment can help many patients improve their QOL. Brain–machine interfaces (BMIs), particularly brain–computer interfaces (BCIs) can help many patients who are in end-stage spinal degeneration or spinal disease [144]. BCIs like those developed by Neuralink utilize high-density microelectrode arrays with over 3000 channels per implant to enable direct bidirectional communication with cortical networks. These implants decode motor intent and, when coupled with spinal stimulation systems, can restore volitional control of paralyzed limbs [145]. Although this field is still experimental, early human studies such as Neuralink’s “Telepathy” interface have shown encouraging results in motor-imagery–based control by quadriplegic users [146]. From a spinal pathology standpoint, these technologies are particularly relevant for patients with cervical spinal cord injury or end-stage degenerative myelopathy, where native neural circuits may be disrupted. Ongoing studies are exploring how closed-loop systems, combining cortical interfaces with epidural or intraspinal stimulation, can bypass damaged pathways and re-establish functional connectivity. While still in early stages, these approaches hold potential as adjunctive therapies when traditional surgical or regenerative interventions offer limited benefit. Neuralink recently raised $650 million in Series E funding to conduct further clinical trials [147]. As its competitors like Synchron, Paradromics, Blackrock Neurotech, and Precision Neuroscience also develop their BCI or BCI-analogous technology, it seems that patients who have mobility difficulty or an inability can soon benefit from new technology. An ideal future platform may blend Piezo-activated neural scaffolds with real-time BMI feedback to support neuromotor control and neural repair.

## 7. Conclusions

The mechanobiology of the spine reflects a complex interplay between molecular signaling, mechanical stress, inflammation, and tissue regeneration. As outlined in this review, core pathways such as integrin-FAK-MAPK, YAP and TAZ signaling, Wnt/β-catenin, and Piezo ion channels regulate spinal tissue homeostasis and respond to both biomechanical and inflammatory cues. These molecular systems govern structural integrity and functional responses in intervertebral discs, vertebrae, ligaments, and neural elements.

Recent advances in regenerative medicine have yielded promising therapeutic platforms. These include stem cell therapies tailored to disc microenvironments, tissue-engineered scaffolds that mimic native biomechanical properties, and neural interfaces that enable real-time feedback for spinal cord injury recovery. Each of these approaches leverages specific mechanobiological pathways to promote repair and restore function in damaged spinal tissues.

Despite these encouraging developments, several challenges remain. The translation of preclinical findings into clinical application requires careful attention to immune compatibility, long-term scaffold integration, and consistent signaling activation. Molecular targets such as IL-6, TNF-alpha, and IL-1 beta remain difficult to modulate without off-target effects, particularly within complex inflammatory environments. Neural interfaces and high-density electrode systems introduce additional concerns related to safety, durability, and ethical oversight.

As research progresses, it is important to balance innovation with rigorous validation. Mechanobiology-informed therapies must be tested in scalable, reproducible systems that reflect the heterogeneity of spinal disorders. Technologies such as bioreactors, epigenetic modulation, and smart biomaterials offer new pathways to refine these interventions.

In conclusion, the integration of molecular insight with biomechanical innovation holds substantial promise for transforming spinal care. While caution is warranted due to the complexity of spinal biology, the careful development of mechanobiology-driven therapies has the potential to deliver personalized, effective, and long-lasting solutions for patients with spinal disease.

This review set out to connect molecular signaling with practical innovations in spine care, tying together basic science with potential applications. Although the topics covered are wide-ranging, the intent was to give readers a structured view of how emerging research fits into the bigger picture—what remains in the experimental stage, what’s gaining clinical traction, and where key knowledge gaps persist. By organizing content around specific spinal tissues and including subheadings to break up dense material, the goal was to make the science easier to follow and highlight the most relevant takeaways for both researchers and clinicians.

## Data Availability

No new data were created or analyzed in this study. Data sharing is not applicable to this article.

## References

[1] Jaalouk D.E., Lammerding J. (2009). Mechanotransduction gone awry. Nat. Rev. Mol. Cell Biol..

[2] Diwan A.D., Melrose J. (2022). Intervertebral disc degeneration and how it leads to low back pain. JOR Spine.

[3] Waxenbaum J.A., Reddy V., Futterman B. (2025). Anatomy, Back, Intervertebral Discs. StatPearls.

[4] Sun Z., Guo S.S., Fässler R. (2016). Integrin-mediated mechanotransduction. J. Cell Biol..

[5] Strohmeyer N., Bharadwaj M., Costell M., Fässler R., Müller D.J. (2017). Fibronectin-bound α5β1 integrins sense load and signal to reinforce adhesion in less than a second. Nat. Mater..

[6] Sawai H., Okada Y., Funahashi H., Matsuo Y., Takahashi H., Takeyama H., Manabe T. (2005). Activation of focal adhesion kinase enhances the adhesion and invasion of pancreatic cancer cells via extracellular signal-regulated kinase-1/2 signaling pathway activation. Mol. Cancer.

[7] Hayes A.J., Melrose J. (2020). Aggrecan, the Primary Weight-Bearing Cartilage Proteoglycan, Has Context-Dependent, Cell-Directive Properties in Embryonic Development and Neurogenesis: Aggrecan Glycan Side Chain Modifications Convey Interactive Biodiversity. Biomolecules.

[8] Neidlinger-Wilke C., Galbusera F., Pratsinis H., Mavrogonatou E., Mietsch A., Kletsas D., Wilke H.-J. (2014). Mechanical loading of the intervertebral disc: From the macroscopic to the cellular level. Eur. Spine J..

[9] Schmidt C., Pommerenke H., Dürr F., Nebe B., Rychly J. (1998). Mechanical stressing of integrin receptors induces enhanced tyrosine phosphorylation of cytoskeletally anchored proteins. J. Biol. Chem..

[10] Katoh K. (2025). Integrin and Its Associated Proteins as a Mediator for Mechano-Signal Transduction. Biomolecules.

[11] Maggi A., Li H., Greer J.R. (2017). Three-dimensional nano-architected scaffolds with tunable stiffness for efficient bone tissue growth. Acta Biomater..

[12] Chan B.P., Leong K.W. (2008). Scaffolding in tissue engineering: General approaches and tissue-specific considerations. Eur. Spine J..

[13] Wang L., You X., Zhang L., Zhang C., Zou W. (2022). Mechanical regulation of bone remodeling. Bone Res..

[14] Park E.J., Myint P.K., Ito A., Appiah M.G., Darkwah S., Kawamoto E., Shimaoka M. (2020). Integrin-Ligand Interactions in Inflammation, Cancer, and Metabolic Disease: Insights into the Multifaceted Roles of an Emerging Ligand Irisin. Front. Cell Dev. Biol..

[15] Hu X., Li J., Fu M., Zhao X., Wang W. (2021). The JAK/STAT signaling pathway: From bench to clinic. Signal Transduct. Target. Ther..

[16] Chavez L., Meguro J., Chen S., de Paiva V.N., Zambrano R., Eterno J.M., Kumar R., Duncan M.R., Benny M., Young K.C. (2021). Circulating extracellular vesicles activate the pyroptosis pathway in the brain following ventilation-induced lung injury. J. Neuroinflamm..

[17] Raman N., Imran S.A.M., Ahmad Amin Noordin K.B., Zaman W.S.W.K., Nordin F. (2022). Mechanotransduction in Mesenchymal Stem Cells (MSCs) Differentiation: A Review. Int. J. Mol. Sci..

[18] Wang H.N., Huang Y.C., Ni G.X. (2020). Mechanotransduction of stem cells for tendon repair. World J. Stem Cells.

[19] Pattnaik A., Sanket A.S., Pradhan S., Sahoo R., Das S., Pany S., Douglas T.E.L., Dandela R., Liu Q., Rajadas J. (2023). Designing of gradient scaffolds and their applications in tissue regeneration. Biomaterials.

[20] Wu X.M., Han W.M., Hou L.Y., Lin D.D., Li J.Y., Lin S.T., Yang J.P., Liao L., Zeng X.A. (2024). Glutenin-chitosan 3D porous scaffolds with tunable stiffness and systematized microstructure for cultured meat model. Int. J. Biol. Macromol..

[21] Kegelman C.D., Collins J.M., Nijsure M.P., Eastburn E.A., Boerckel J.D. (2020). Gone Caving: Roles of the Transcriptional Regulators YAP and TAZ in Skeletal Development. Curr. Osteoporos. Rep..

[22] Heng B.C., Zhang X., Aubel D., Bai Y., Li X., Wei Y., Fussenegger M., Deng X. (2021). An overview of signaling pathways regulating YAP/TAZ activity. Cell. Mol. Life Sci..

[23] Zhu J., Wu T., Lin Q. (2023). Non-hippo kinases: Indispensable roles in YAP/TAZ signaling and implications in cancer therapy. Mol. Biol. Rep..

[24] Han J., Zhang J., Zhang X., Luo W., Liu L., Zhu Y., Liu Q., Zhang X.A. (2024). Emerging role and function of Hippo-YAP/TAZ signaling pathway in musculoskeletal disorders. Stem Cell Res. Ther..

[25] Thampatty B.P., Li H., Im H.J., Wang J.H. (2007). EP4 receptor regulates collagen type-I, MMP-1, and MMP-3 gene expression in human tendon fibroblasts in response to IL-1 beta treatment. Gene.

[26] Kegelman C.D., Coulombe J.C., Jordan K.M., Horan D.J., Qin L., Robling A.G., Ferguson V.L., Bellido T.M., Boerckel J.D. (2020). YAP and TAZ Mediate Osteocyte Perilacunar/Canalicular Remodeling. J. Bone Miner. Res..

[27] Shin S.M., Moehring F., Itson-Zoske B., Fan F., Stucky C.L., Hogan Q.H., Yu H. (2021). Piezo2 mechanosensitive ion channel is located to sensory neurons and nonneuronal cells in rat peripheral sensory pathway: Implications in pain. Pain.

[28] Woo S.H., Lukacs V., de Nooij J.C., Zaytseva D., Criddle C.R., Francisco A., Jessell T.M., Wilkinson K.A., Patapoutian A. (2015). Piezo2 is the principal mechanotransduction channel for proprioception. Nat. Neurosci..

[29] Liu H., Hu J., Zheng Q., Feng X., Zhan F., Wang X., Xu G., Hua F. (2022). Piezo1 Channels as Force Sensors in Mechanical Force-Related Chronic Inflammation. Front. Immunol..

[30] Yu L., Tian D., Su Z., Zhang L., Jie L., Guo S., Zhu W., Zhang N., Wang P. (2025). Mechanical stress overload promotes NF-κB/NLRP3-mediated osteoarthritis synovitis and fibrosis through Piezo1. Cell. Signal..

[31] Aragona M., Mhalhel K., Pansera L., Montalbano G., Guerrera M.C., Levanti M., Laurà R., Abbate F., Vega J.A., Germanà A. (2024). Localization of Piezo 1 and Piezo 2 in Lateral Line System and Inner Ear of Zebrafish (*Danio rerio*). Int. J. Mol. Sci..

[32] Jiang D., Zhao J., Zheng J., Zhao Y., Le M., Qin D., Huang Q., Huang J., Zhao Q., Wang L. (2024). LOX-mediated ECM mechanical stress induces Piezo1 activation in hypoxic-ischemic brain damage and identification of novel inhibitor of LOX. Redox Biol..

[33] Garcia-Castorena J.M., Riester R., Gamino-Ornelas M., Ada N., Guilak F., Danalache M. (2025). PIEZO1-mediated calcium influx transiently alters nuclear mechanical properties via actin remodeling in chondrocytes. Biochem. Biophys. Res. Commun..

[34] Eijkelkamp N., Linley J.E., Torres J.M., Bee L., Dickenson A.H., Gringhuis M., Minett M.S., Hong G.S., Lee E., Oh U. (2013). A role for Piezo2 in EPAC1-dependent mechanical allodynia. Nat. Commun..

[35] Obeidat A.M., Wood M.J., Adamczyk N.S., Ishihara S., Li J., Wang L., Ren D., Bennett D.A., Miller R.J., Malfait A.M. (2023). Piezo2 expressing nociceptors mediate mechanical sensitization in experimental osteoarthritis. Nat. Commun..

[36] Kumar R., Sporn K., Borole A., Khanna A., Gowda C., Paladugu P., Ngo A., Jagadeesan R., Zaman N., Tavakkoli A. (2025). Biomarker-Guided Imaging and AI-Augmented Diagnosis of Degenerative Joint Disease. Diagnostics.

[37] Xie Z., Feng J., Hibberd T.J., Chen B.N., Zhao Y., Zang K., Hu X., Yang X., Chen L., Brookes S.J. (2023). Piezo2 channels expressed by colon-innervating TRPV1-lineage neurons mediate visceral mechanical hypersensitivity. Neuron.

[38] Murthy S.E., Loud M.C., Daou I., Marshall K.L., Schwaller F., Kühnemund J., Francisco A.G., Keenan W.T., Dubin A.E., Lewin G.R. (2018). The mechanosensitive ion channel Piezo2 mediates sensitivity to mechanical pain in mice. Sci. Transl. Med..

[39] Wan Y., Zhou J., Li H. (2024). The Role of Mechanosensitive Piezo Channels in Chronic Pain. J. Pain Res..

[40] MacDonald B.T., Tamai K., He X. (2009). Wnt/beta-catenin signaling: Components, mechanisms, and diseases. Dev. Cell.

[41] Devos H., Zoidakis J., Roubelakis M.G., Latosinska A., Vlahou A. (2023). Reviewing the Regulators of COL1A1. Int. J. Mol. Sci..

[42] Wadhwa H., Rohde M., Koltsov J.C.B., Cabell A., Smuck M., Hu S.S., Kleimeyer J.P. (2025). Incidence and risk factors for complications following cervical epidural steroid injections. Spine J..

[43] Hiyama A., Sakai D., Risbud M.V., Tanaka M., Arai F., Abe K., Mochida J. (2010). Enhancement of intervertebral disc cell senescence by WNT/β-catenin signaling-induced matrix metalloproteinase expression. Arthritis Rheum..

[44] Hu L., Chen W., Qian A., Li Y.P. (2024). Wnt/β-catenin signaling components and mechanisms in bone formation, homeostasis, and disease. Bone Res..

[45] Premaraj S., Souza I., Premaraj T. (2011). Mechanical loading activates β-catenin signaling in periodontal ligament cells. Angle Orthod..

[46] Tigchelaar S.S., Wadhwa H., Mathur M.B., He Z., Tharin S. (2023). Spinal cord injury: A systematic review and meta-analysis of microRNA alterations. BioRxiv.

[47] Bastakoty D., Saraswati S., Cates J., Lee E., Nanney L.B., Young P.P. (2015). Inhibition of Wnt/β-catenin pathway promotes regenerative repair of cutaneous and cartilage injury. FASEB J..

[48] Ravi K., Paidas M.J., Saad A., Jayakumar A.R. (2021). Astrocytes in rare neurological conditions: Morphological and functional considerations. J. Comp. Neurol..

[49] Mahajan A., Nengroo M.A., Datta D., Katti D.S. (2023). Converse modulation of Wnt/β-catenin signaling during expansion and differentiation phases of Infrapatellar fat pad-derived MSCs for improved engineering of hyaline cartilage. Biomaterials.

[50] Tong L., Yu H., Huang X., Shen J., Xiao G., Chen L., Wang H., Xing L., Chen D. (2022). Current understanding of osteoarthritis pathogenesis and relevant new approaches. Bone Res..

[51] Suryawanshi A., Tadagavadi R.K., Swafford D., Manicassamy S. (2016). Modulation of Inflammatory Responses by Wnt/β-Catenin Signaling in Dendritic Cells: A Novel Immunotherapy Target for Autoimmunity and Cancer. Front. Immunol..

[52] Yin Z., Guo J., Wu T.Y., Chen X., Xu L.L., Lin S.E., Sun Y.X., Chan K.M., Ouyang H., Li G. (2016). Stepwise Differentiation of Mesenchymal Stem Cells Augments Tendon-Like Tissue Formation and Defect Repair In Vivo. Stem Cells Transl. Med..

[53] Brown P.T., Handorf A.M., Jeon W.B., Li W.J. (2013). Stem cell-based tissue engineering approaches for musculoskeletal regeneration. Curr. Pharm. Des..

[54] Sakai T., Kumagai K. (2025). Molecular dissection of tendon development and healing: Insights into tenogenic phenotypes and functions. J. Biol. Chem..

[55] Yilgor C., Yilgor Huri P., Huri G. (2012). Tissue engineering strategies in ligament regeneration. Stem Cells Int..

[56] Hoang V.T., Nguyen Q.T., Phan T.T.K., Pham T.H., Dinh N.T.H., Anh L.P.H., Dao L.T.M., Bui V.D., Dao H.N., Le D.S. (2025). Tissue Engineering and Regenerative Medicine: Perspectives and Challenges. MedComm.

[57] Ajalik R.E., Alenchery R.G., Cognetti J.S., Zhang V.Z., McGrath J.L., Miller B.L., Awad H.A. (2022). Human Organ-on-a-Chip Microphysiological Systems to Model Musculoskeletal Pathologies and Accelerate Therapeutic Discovery. Front. Bioeng. Biotechnol..

[58] Kishimoto Y., Ohkawara B., Sakai T., Ito M., Masuda A., Ishiguro N., Shukunami C., Docheva D., Ohno K. (2017). Wnt/β-catenin signaling suppresses expressions of Scx, Mkx, and Tnmd in tendon-derived cells. PLoS ONE.

[59] Ciardulli M.C., Marino L., Lovecchio J., Giordano E., Forsyth N.R., Selleri C., Maffulli N., Porta G.D. (2020). Tendon and Cytokine Marker Expression by Human Bone Marrow Mesenchymal Stem Cells in a Hyaluronate/Poly-Lactic-Co-Glycolic Acid (PLGA)/Fibrin Three-Dimensional (3D) Scaffold. Cells.

[60] Wiegertjes R., van de Loo F.A.J., Blaney Davidson E.N. (2020). A roadmap to target interleukin-6 in osteoarthritis. Rheumatology.

[61] Tateiwa D., Yoshikawa H., Kaito T. (2019). Cartilage and Bone Destruction in Arthritis: Pathogenesis and Treatment Strategy: A Literature Review. Cells.

[62] Wang Y., van Boxel-Dezaire A.H., Cheon H., Yang J., Stark G.R. (2013). STAT3 activation in response to IL-6 is prolonged by the binding of IL-6 receptor to EGF receptor. Proc. Natl. Acad. Sci. USA.

[63] Taylor P.C., Feist E., Pope J.E., Nash P., Sibilia J., Caporali R., Balsa A. (2024). What have we learnt from the inhibition of IL-6 in RA and what are the clinical opportunities for patient outcomes?. Ther. Adv. Musculoskelet. Dis..

[64] Ferrari L.F., Khomula E.V., Araldi D., Levine J.D. (2018). CD44 Signaling Mediates High Molecular Weight Hyaluronan-Induced Antihyperalgesia. J. Neurosci..

[65] Choi H., Yeo M., Kang Y., Kim H.J., Park S.G., Jang E., Park S.H., Kim E., Kang S. (2023). Lactate oxidase/catalase-displaying nanoparticles efficiently consume lactate in the tumor microenvironment to effectively suppress tumor growth. J. Nanobiotechnol..

[66] Liu N., Wang X., Wang Z., Kan Y., Fang Y., Gao J., Kong X., Wang J. (2025). Nanomaterials-driven in situ vaccination: A novel frontier in tumor immunotherapy. J. Hematol. Oncol..

[67] Feng Y., Tang Q., Wang B., Yang Q., Zhang Y., Lei L., Li S. (2024). Targeting the tumor microenvironment with biomaterials for enhanced immunotherapeutic efficacy. J. Nanobiotechnol..

[68] Moazzam M., Zhang M., Hussain A., Yu X., Huang J., Huang Y. (2024). The landscape of nanoparticle-based siRNA delivery and therapeutic development. Mol. Ther..

[69] Johnson Z.I., Schoepflin Z.R., Choi H., Shapiro I.M., Risbud M.V. (2015). Disc in flames: Roles of TNF-α and IL-1β in intervertebral disc degeneration. Eur. Cells Mater..

[70] Choi M.C., Jo J., Park J., Kang H.K., Park Y. (2019). NF-κB Signaling Pathways in Osteoarthritic Cartilage Destruction. Cells.

[71] Guo Q., Jin Y., Chen X., Ye X., Shen X., Lin M., Zeng C., Zhou T., Zhang J. (2024). NF-κB in biology and targeted therapy: New insights and translational implications. Signal Transduct. Target. Ther..

[72] Haseeb A., Chen D., Haqqi T.M. (2013). Delphinidin inhibits IL-1β-induced activation of NF-κB by modulating the phosphorylation of IRAK-1(Ser376) in human articular chondrocytes. Rheumatology.

[73] Velnar T., Gradisnik L. (2023). Endplate role in the degenerative disc disease: A brief review. World J. Clin. Cases.

[74] Kievit W., Fransen J., Oerlemans A.J., Kuper H.H., van der Laar M.A., de Rooij D.J., De Gendt C.M., Ronday K.H., Jansen T.L., van Oijen P.C. (2007). The efficacy of anti-TNF in rheumatoid arthritis, a comparison between randomised controlled trials and clinical practice. Ann. Rheum. Dis..

[75] Lee N.J., Ha S.K., Sati P., Absinta M., Nair G., Luciano N.J., Leibovitch E.C., Yen C.C., Rouault T.A., Silva A.C. (2019). Potential role of iron in repair of inflammatory demyelinating lesions. J. Clin. Investig..

[76] Donadieu M., Lee N.J., Gaitán M.I., Ha S.K., Luciano N.J., Roy S., Ineichen B., Leibovitch E.C., Yen C.C., Pham D.L. (2023). In vivo MRI is sensitive to remyelination in a nonhuman primate model of multiple sclerosis. eLife.

[77] Brovold M., Almeida J.I., Pla-Palacín I., Sainz-Arnal P., Sánchez-Romero N., Rivas J.J., Almeida H., Dachary P.R., Serrano-Aulló T., Soker S. (2018). Naturally-Derived Biomaterials for Tissue Engineering Applications. Adv. Exp. Med. Biol..

[78] Fortier L.A., Barker J.U., Strauss E.J., McCarrel T.M., Cole B.J. (2011). The role of growth factors in cartilage repair. Clin. Orthop. Relat. Res..

[79] Wang M., Wang J., Xu X., Li E., Xu P. (2024). Engineering gene-activated bioprinted scaffolds for enhancing articular cartilage repair. Mater. Today Bio.

[80] Kacprzak B., Stańczak M., Bielenda B., Yarmohammadi A.A., Hagner-Derengowska M. (2025). Molecular Aspects of Cartilage Microfracturation: Rehabilitation Insights. Orthop. Rev..

[81] McClurg O., Tinson R., Troeberg L. (2021). Targeting Cartilage Degradation in Osteoarthritis. Pharmaceuticals.

[82] Tolstova T., Dotsenko E., Luzgina N., Rusanov A. (2024). Preconditioning of Mesenchymal Stem Cells Enhances the Neuroprotective Effects of Their Conditioned Medium in an Alzheimer’s Disease In Vitro Model. Biomedicines.

[83] Haider K.H. (2024). Priming mesenchymal stem cells to develop “super stem cells”. World J. Stem Cells.

[84] Yang H., Yang H., Wang Q., Ji H., Qian T., Qiao Y., Shi J., Cong M. (2025). Mesenchymal stem cells and their extracellular vesicles: New therapies for cartilage repair. Front. Bioeng. Biotechnol..

[85] Paladugu P., Kumar R., Hage T., Vaja S., Sekhar T., Weisberg S., Sporn K., Waisberg E., Ong J., Vadhera A. (2025). Leveraging lower body negative pressure for enhanced outcomes in orthopedic arthroplasty—Insights from NASA’s bone health research. Life Sci. Space Res..

[86] Srinivasan D., Yen J.H., Joseph D.J., Friedman W. (2004). Cell type-specific interleukin-1beta signaling in the CNS. J. Neurosci..

[87] Zhao Z., Wang Y., Zhou R., Li Y., Gao Y., Tu D., Wilson B., Song S., Feng J., Hong J.-S. (2020). A novel role of NLRP3-generated IL-1β in the acute-chronic transition of peripheral lipopolysaccharide-elicited neuroinflammation: Implications for sepsis-associated neurodegeneration. J. Neuroinflamm..

[88] Vannini F., Kashfi K., Nath N. (2015). The dual role of iNOS in cancer. Redox Biol..

[89] Cota Quintero J.L., Ramos-Payán R., Romero-Quintana J.G., Ayala-Ham A., Bermúdez M., Aguilar-Medina E.M. (2025). Hydrogel-Based Scaffolds: Advancing Bone Regeneration Through Tissue Engineering. Gels.

[90] Li W., Hu J., Chen C., Li X., Zhang H., Xin Y., Tian Q., Wang S. (2023). Emerging advances in hydrogel-based therapeutic strategies for tissue regeneration. Regen. Ther..

[91] Chen R., Chen F., Chen K., Xu J. (2023). Advances in the application of hydrogel-based scaffolds for tendon repair. Genes Dis..

[92] Cai Y., Liu Z., Wang H., Meng H., Cao Y. (2024). Mesoporous Silica Nanoparticles Mediate SiRNA Delivery for Long-Term Multi-Gene Silencing in Intact Plants. Adv. Sci..

[93] Li Y., Deng G., Hu X., Li C., Wang X., Zhu Q., Zheng K., Xiong W., Wu H. (2022). Recent advances in mesoporous silica nanoparticle-based targeted drug-delivery systems for cancer therapy. Nanomedicine.

[94] Khaliq N.U., Lee J., Kim J., Kim Y., Yu S., Kim J., Kim S., Sung D., Kim H. (2023). Mesoporous Silica Nanoparticles as a Gene Delivery Platform for Cancer Therapy. Pharmaceutics.

[95] Saparov A., Ogay V., Nurgozhin T., Jumabay M., Chen W.C. (2016). Preconditioning of Human Mesenchymal Stem Cells to Enhance Their Regulation of the Immune Response. Stem Cells Int..

[96] Xue P., Wang Y., Lv L., Wang D., Wang Y. (2024). Roles of Chemokines in Intervertebral Disk Degeneration. Curr. Pain Headache Rep..

[97] Hicks M.R., Cao T.V., Campbell D.H., Standley P.R. (2012). Mechanical strain applied to human fibroblasts differentially regulates skeletal myoblast differentiation. J. Appl. Physiol..

[98] Shi C., Pamer E.G. (2011). Monocyte recruitment during infection and inflammation. Nat. Rev. Immunol..

[99] Bess S.N., Greening G.J., Rajaram N., Muldoon T.J. (2022). Macrophage-targeted anti-CCL2 immunotherapy enhances tumor sensitivity to 5-fluorouracil in a Balb/c-CT26 murine colon carcinoma model measured using diffuse reflectance spectroscopy. BMC Immunol..

[100] Sullivan C.B., Porter R.M., Evans C.H., Ritter T., Shaw G., Barry F., Murphy J.M. (2014). TNFα and IL-1β influence the differentiation and migration of murine MSCs independently of the NF-κB pathway. Stem Cell Res. Ther..

[101] Katanov C., Lerrer S., Liubomirski Y., Leider-Trejo L., Meshel T., Bar J., Feniger-Barish R., Kamer I., Soria-Artzi G., Kahani H. (2015). Regulation of the inflammatory profile of stromal cells in human breast cancer: Prominent roles for TNF-α and the NF-κB pathway. Stem Cell Res Ther..

[102] Kuang P.P., Liu X.Q., Li C.G., He B.X., Xie Y.C., Wu Z.C., Li C.L., Deng X.H., Fu Q.L. (2023). Mesenchymal stem cells overexpressing interleukin-10 prevent allergic airway inflammation. Stem Cell Res. Ther..

[103] Diekman B.O., Wu C.L., Louer C.R., Furman B.D., Huebner J.L., Kraus V.B., Olson S.A., Guilak F. (2013). Intra-articular delivery of purified mesenchymal stem cells from C57BL/6 or MRL/MpJ superhealer mice prevents posttraumatic arthritis. Cell Transplant..

[104] Sukubo N.G., Bigini P., Morelli A. (2025). Nanocarriers and macrophage interaction: From a potential hurdle to an alternative therapeutic strategy. Beilstein J. Nanotechnol..

[105] Wong V.W., Akaishi S., Longaker M.T., Gurtner G.C. (2011). Pushing back: Wound mechanotransduction in repair and regeneration. J. Investig. Dermatol..

[106] Wang L., Zhang J., Qiu S., Huang R., Wang Y., Wang Y., Li M., Ye Q., Zhang S., Qi Z. (2025). IL-33/ST2 drives inflammatory pain via CCL2 signaling and activation of TRPV1 and TRPM8. Commun. Biol..

[107] Kumar R., Sporn K., Paladugu P., Khanna A., Gowda C., Ngo A., Waisberg E., Ong J., Jagadeesan R., Tavakkoli A. (2025). Emerging Diagnostic Approaches for Musculoskeletal Disorders: Advances in Imaging, Biomarkers, and Clinical Assessment. Diagnostics.

[108] Li Q., Yang Z., Wang K., Chen Z., Shen H. (2023). Suppression of microglial Ccl2 reduces neuropathic pain associated with chronic spinal compression. Front. Immunol..

[109] Kumar R., Sporn K., Gowda C., Khanna A., Prabhakar P., Paladugu P., Jagadeesan R., Clarkson L., Chandrahasegowda S., Kumar T. (2025). Advancing Spine Connectomics and Neural Integration through Machine Learning and Neuroengineering. Preprints.

[110] Page-McCaw A., Ewald A.J., Werb Z. (2007). Matrix metalloproteinases and the regulation of tissue remodelling. Nat. Rev. Mol. Cell Biol..

[111] Nakao A., Imamura T., Souchelnytskyi S., Kawabata M., Ishisaki A., Oeda E., Tamaki K., Hanai J., Heldin C.H., Miyazono K. (1997). TGF-beta receptor-mediated signalling through Smad2, Smad3 and Smad4. EMBO J..

[112] Verrecchia F., Mauviel A. (2002). Transforming growth factor-beta signaling through the Smad pathway: Role in extracellular matrix gene expression and regulation. J. Investig. Dermatol..

[113] Song N., Scholtemeijer M., Shah K. (2020). Mesenchymal Stem Cell Immunomodulation: Mechanisms and Therapeutic Potential. Trends Pharmacol. Sci..

[114] Lin T., Pajarinen J., Nabeshima A., Lu L., Nathan K., Yao Z., Goodman S.B. (2017). Establishment of NF-κB sensing and interleukin-4 secreting mesenchymal stromal cells as an “on-demand” drug delivery system to modulate inflammation. Cytotherapy.

[115] Hoshi H., Akagi R., Yamaguchi S., Muramatsu Y., Akatsu Y., Yamamoto Y., Sasaki T., Takahashi K., Sasho T. (2017). Effect of inhibiting MMP13 and ADAMTS5 by intra-articular injection of small interfering RNA in a surgically induced osteoarthritis model of mice. Cell Tissue Res..

[116] Zhang Y., Pizzute T., Pei M. (2014). Anti-inflammatory strategies in cartilage repair. Tissue Eng. Part B Rev..

[117] Xie X., Yue T., Gu W., Cheng W., He L., Ren W., Li F., Piao J.G. (2023). Recent Advances in Mesoporous Silica Nanoparticles Delivering siRNA for Cancer Treatment. Pharmaceutics.

[118] Yap K.M., Sekar M., Fuloria S., Wu Y.S., Gan S.H., Mat Rani N.N.I., Subramaniyan V., Kokare C., Lum P.T., Begum M.Y. (2021). Drug Delivery of Natural Products Through Nanocarriers for Effective Breast Cancer Therapy: A Comprehensive Review of Literature. Int. J. Nanomed..

[119] Desai P.R., Marepally S., Patel A.R., Voshavar C., Chaudhuri A., Singh M. (2013). Topical delivery of anti-TNFα siRNA and capsaicin via novel lipid-polymer hybrid nanoparticles efficiently inhibits skin inflammation in vivo. J. Control. Release.

[120] Chan C.M., Macdonald C.D., Litherland G.J., Wilkinson D.J., Skelton A., Europe-Finner G.N., Rowan A.D. (2017). Cytokine-induced MMP13 Expression in Human Chondrocytes Is Dependent on Activating Transcription Factor 3 (ATF3) Regulation. J. Biol. Chem..

[121] Hu Q., Ecker M. (2021). Overview of MMP-13 as a Promising Target for the Treatment of Osteoarthritis. Int. J. Mol. Sci..

[122] Tian Y., Yuan W., Fujita N., Wang J., Wang H., Shapiro I.M., Risbud M.V. (2013). Inflammatory cytokines associated with degenerative disc disease control aggrecanase-1 (ADAMTS-4) expression in nucleus pulposus cells through MAPK and NF-κB. Am. J. Pathol..

[123] Zeng Q., Sun Q., Xu H., Chen J., Ling H., Ge Q., Zou K., Wang X., Jin H., Li J. (2023). Amygdalin Delays Cartilage Endplate Degeneration and Improves Intervertebral Disc Degeneration by Inhibiting NF-κB Signaling Pathway and Inflammatory Response. J. Inflamm. Res..

[124] Tabeian H., Betti B.F., Dos Santos Cirqueira C., de Vries T.J., Lobbezoo F., Ter Linde A.V., Zandieh-Doulabi B., Koenders M.I., Everts V., Bakker A.D. (2019). IL-1β Damages Fibrocartilage and Upregulates MMP-13 Expression in Fibrochondrocytes in the Condyle of the Temporomandibular Joint. Int. J. Mol. Sci..

[125] Masuda K., Lotz J.C. (2010). New challenges for intervertebral disc treatment using regenerative medicine. Tissue Eng. Part B Rev..

[126] Zhang J., Sun T., Zhang W., Yang M., Li Z. (2022). Autologous cultured adipose derived mesenchymal stem cells combined with hyaluronic acid hydrogel in the treatment of discogenic low back pain: A study protocol for a phase II randomised controlled trial. BMJ Open.

[127] Clarke L.E., McConnell J.C., Sherratt M.J., Derby B., Richardson S.M., Hoyland J.A. (2014). Growth differentiation factor 6 and transforming growth factor-beta differentially mediate mesenchymal stem cell differentiation, composition, and micromechanical properties of nucleus pulposus constructs. Arthritis Res. Ther..

[128] Wei A., Shen B., Williams L., Diwan A. (2014). Mesenchymal stem cells: Potential application in intervertebral disc regeneration. Transl. Pediatr..

[129] Yang Y., Lee E.H., Yang Z. (2022). Hypoxia-Conditioned Mesenchymal Stem Cells in Tissue Regeneration Application. Tissue Eng. Part B Rev..

[130] Nakamura N., Shi X., Darabi R., Li Y. (2021). Hypoxia in Cell Reprogramming and the Epigenetic Regulations. Front. Cell Dev. Biol..

[131] Camuzi D., de Amorim Í.S.S., Ribeiro Pinto L.F., Oliveira Trivilin L., Mencalha A.L., Soares Lima S.C. (2019). Regulation Is in the Air: The Relationship between Hypoxia and Epigenetics in Cancer. Cells.

[132] Lu P., Ruan D., Huang M., Tian M., Zhu K., Gan Z., Xiao Z. (2024). Harnessing the potential of hydrogels for advanced therapeutic applications: Current achievements and future directions. Signal Transduct. Target. Ther..

[133] Yang C., Cai W., Xiang P., Liu Y., Xu H., Zhang W., Han F., Luo Z., Liang T. (2024). Viscoelastic hydrogel combined with dynamic compression promotes osteogenic differentiation of bone marrow mesenchymal stem cells and bone repair in rats. Regen. Biomater..

[134] Peffers M.J., Thorpe C.T., Collins J.A., Eong R., Wei T.K., Screen H.R., Clegg P.D. (2014). Proteomic analysis reveals age-related changes in tendon matrix composition, with age- and injury-specific matrix fragmentation. J. Biol. Chem..

[135] Hade M.D., Suire C.N., Suo Z. (2021). Mesenchymal Stem Cell-Derived Exosomes: Applications in Regenerative Medicine. Cells.

[136] Wang W., Yeung K.W.K. (2017). Bone grafts and biomaterials substitutes for bone defect repair: A review. Bioact. Mater..

[137] Gillespie E.F., Santos P.M.G., Curry M., Salz T., Chakraborty N., Caron M., Fuchs H.E., Vicioso N.L., Mathis N., Kumar R. (2024). Implementation Strategies to Promote Short-Course Radiation for Bone Metastases. JAMA Netw. Open.

[138] Kegelman C.D., Nijsure M.P., Moharrer Y., Pearson H.B., Dawahare J.H., Jordan K.M., Qin L., Boerckel J.D. (2021). YAP and TAZ Promote Periosteal Osteoblast Precursor Expansion and Differentiation for Fracture Repair. J. Bone Miner. Res..

[139] Wang R.N., Green J., Wang Z., Deng Y., Qiao M., Peabody M., Zhang Q., Ye J., Yan Z., Denduluri S. (2014). Bone Morphogenetic Protein (BMP) signaling in development and human diseases. Genes Dis..

[140] Binaymotlagh R., Chronopoulou L., Palocci C. (2023). Peptide-Based Hydrogels: Template Materials for Tissue Engineering. J. Funct. Biomater..

[141] Yao Q., Sandhurst E.S., Liu Y., Sun H. (2017). BBP-Functionalized Biomimetic Nanofibrous Scaffold Can Capture BMP2 and Promote Osteogenic Differentiation. J. Mater. Chem. B.

[142] Barakat H., Aljutaily T., Almujaydil M.S., Algheshairy R.M., Alhomaid R.M., Almutairi A.S., Alshimali S.I., Abdellatif A.A.H. (2022). Amygdalin: A Review on Its Characteristics, Antioxidant Potential, Gastrointestinal Microbiota Intervention, Anticancer Therapeutic and Mechanisms, Toxicity, and Encapsulation. Biomolecules.

[143] Li K., Han J., Wang Z. (2021). Histone modifications centric-regulation in osteogenic differentiation. Cell Death Discov..

[144] Kumar R., Waisberg E., Ong J., Lee A.G. (2025). The potential power of Neuralink-how brain-machine interfaces can revolutionize medicine. Expert Rev. Med. Devices.

[145] Musk E., Neuralink (2019). An Integrated Brain-Machine Interface Platform with Thousands of Channels. J. Med. Internet Res..

[146] Ognard J., El Hajj G., Verma O., Ghozy S., Kadirvel R., Kallmes D.F., Brinjikji W. (2025). Advances in endovascular brain computer interface: Systematic review and future implications. J. Neurosci. Methods.

[147] Neuralink (2025). Neuralink Raises $650 Million Series E. https://neuralink.com/updates/neuralink-raises-650m-series-e/.

